# Optimization of Extraction and Refining Parameters of Oil from Dotted Gizzard Shad (*Konosirus punctatus*)

**DOI:** 10.3390/foods13081278

**Published:** 2024-04-22

**Authors:** Ying Guo, Juanjuan Shao, Jilu Sun, Zhen Wang, Baojie Jiang

**Affiliations:** 1 College of Science and Technology, Hebei Agricultural University, Cangzhou 061100, China; gying0009@163.com (Y.G.); wangzhen@hebau.edu.cn (Z.W.); lgjbj@hebau.edu.cn (B.J.); 2 College of Food Science and Technology, Hebei Agricultural University, Baoding 071001, China; shpsjl@hebau.edu.cn

**Keywords:** *Konosirus punctatus*, fish oil, ethanol-assisted enzymatic hydrolysis, refined oil

## Abstract

To address the challenges associated with resource inefficiency, low extraction rates, environmental concerns, and high energy consumption in traditional fish oil production from dotted gizzard shad (*Konosirus punctatus*), a novel approach is needed. This study aimed to develop and evaluate two innovative methods for fish oil extraction and refinement, focusing on their effects on fish oil quality, fatty acid profile, and volatile compound composition throughout the respective processes. The findings of the study revealed that the ethanol-assisted enzymatic extraction method surpassed the conventional enzymatic approach in extraction efficiency, achieving an optimal extraction rate of 74.94% ± 0.45% under optimized process conditions. Moreover, the ethanol-NaOH one-step degumming and deacidification method proved effective in simultaneously removing phospholipids and free fatty acids. Under optimal conditions, a notable reduction in phospholipid content in dotted gizzard shad oil, from 6.80 ± 0.01 mg/g to 1.18 ± 0.01 mg/g, and a substantial decrease in acid value, from 3.31 mg/g to 0.31 mg/g, were observed. In summary, the study analyzed the physicochemical properties, fatty acid composition, and volatile components of fish oil before and after refinement. The refining process was found to preserve the fatty acid composition while efficiently eliminating hydroperoxides and reducing unpleasant odors in the crude oil.

## 1. Introduction

As living standards improve, individuals are increasingly prioritizing their health, leading to a rising demand for healthcare products and services. Fish oil, derived from fish processing, is notable for its high content of unsaturated fatty acids, particularly EPA and DHA, renowned for their health benefits. These fatty acids constitute a significant portion of the oil, sparking widespread interest across various sectors [1]. Dotted gizzard shad (*Konosirus punctatus*), a prevalent and economically significant small fish in China’s coastal regions, inhabits shallow waters. It is widely distributed across the Bohai Sea, Yellow Sea, East China Sea, and South China Sea [2]. Despite being commonly available in the market at a low price, significantly lower than other fish typically used for fish oil extraction, dotted gizzard shad possesses high regenerative capacity and abundance, safeguarding it from the risk of depletion due to overfishing. However, despite its low market value, the small size, presence of small bony spines, poor texture, and high fat content of dotted gizzard shad make it less suitable as a primary source for fish oil production [3]. Consequently, the added value of dotted gizzard shad products is minimal, inevitably reducing its economic worth.

Fish oil extraction can be achieved through either mechanical or chemical methods. Mechanical methods are generally considered more environmentally friendly, whereas chemical methods typically yield higher extraction rates. In industrial production, conventional methods for obtaining fish oil include pressing, steaming, organic solvents, and enzymolysis [4]. However, the low productivity of the pressing method and the high energy consumption of the steaming method are non-negligible disadvantages of these methods [5]. During enzymatic processing in fish, protein-coated oils and fats are released, leading to an increased extraction rate. In recent years, enzymatic extraction has demonstrated effective performance in extracting fish oils from various sources such as sturgeon oil [6], sea herring oil [7], and tuna viscera oil [8]. On the other hand, the organic solvent method is the traditional approach for fish oil production [9], known for its ease of operation, affordability, and extensive exploration of different extraction reagents. However, the enzymatic process may result in the formation of an emulsified layer, leading to incomplete separation of oils and fats [10]. Organic reagent methods use large amounts of organic solvents, which can potentially leave solvent residues, posing a persistent challenge in modern industrial processes [11]. A combination of organic solvents and enzymatic methods has been used by researchers for the extraction of various substances [10,12,13], and the findings have indicated that this approach can yield favorable extraction rates. Hence, employing non-toxic organic solvents to address the issue of an emulsified layer following enzymatic digestion can effectively mitigate the respective drawbacks of both methods. Crude fish oil typically exhibits a dark color, a fishy odor, and a higher impurity content, which can compromise its stability and impact its color, flavor, and other sensory attributes. Consequently, the quality of fish oil may not fully meet consumption standards [14]. Therefore, impurities need to be removed from crude fish oil to produce fish oil that is suitable for consumption in terms of sensory attributes and quality. The refining procedure typically encompasses several steps, including degumming, deacidification, bleaching, and deodorization. Degumming is aimed at eliminating impurities such as phospholipids, colloids, and proteins [15]. The common method of degumming involves the use of phosphoric acid, which is favored for its low cost and simplicity. However, the requirement for high temperatures and the resultant production of wastewater are significant drawbacks that cannot be overlooked in this traditional degumming approach. The objective of deacidification is to eliminate the free fatty acids present in fish oil. A common method used in traditional processes involves neutralizing the free fatty acids with alkali to reduce the acidity of the fish oil [16]. This process produces saponifications, and a large amount of water is required to remove these saponifications. The currently reported desaponification and deacidification temperatures are high [5,17], which can lead to the oxidation of fatty acids and reduce the quality of fish oil.

The objective of this study was to introduce an ethanol-assisted enzymatic extraction method and an ethanol-NaOH one-step degumming and deacidification method. The aim was to optimize the processes of both methods to substantially enhance the extraction rate of dotted gizzard shad oil and enhance its quality in a manner that is environmentally friendly and energy efficient.

## 2. Materials and Methods

### 2.1. Raw Materials and Chemicals

The raw material used for fish oil extraction was whole dotted gizzard shad (*Konosirus punctatus*), which was purchased and stored at −20 °C until use. Alkaline protease with a potency of 200,000 U/g was obtained from Shenzhen Kangchu Yuan Co., Ltd. (Shenzhen, China). Trypsin (250,000 U/g) and neutral protease (50,000 U/g) were procured from Shanghai Macklin Biochemical Co., Ltd. (Shanghai, China). All other chemicals utilized in this experiment were analytically pure and purchased from Tianjin Komeo Chemical Reagents Co. (Tianjin, China), Beijing Tongguang Fine Chemical Co. (Beijing, China), and Tianjin Dongli District Tianda Chemical Reagent Factory (Tianjin, China).

### 2.2. Analysis of Nutritional Components in Dotted Gizzard Shad

The raw materials were thawed at 4 °C for 12–18 h, then chopped into roughly 2 cm cubes and ground into a mince using a meat grinder. The contents of protein, moisture, fat, and ash in the sample were analyzed. The protein content in the dotted gizzard shad was measured using the second spectrophotometric method according to the Chinese National Standards GB/T 5009.5-2016 [18]. The moisture content was determined using the first method of direct drying in accordance with GB/T 5009.3-2016 [19]. The fat content was assessed using the first method of Soxhlet extraction following GB/T 5009.6-2016 [20], and the ash content was determined by the first method of total ash determination according to GB/T 5009.4-2016 [21].

### 2.3. Fish Oil Extraction

The dotted gizzard shad mince was mixed with distilled water following a specific liquid-to-solid ratio (0.5–2.5 g/g). The pH level of the mixture was adjusted using either hydrochloric acid or sodium hydroxide (2 mol/L) as measured by a pH meter (Starter2C, Shanghai Ohaus Instrument Co., Shanghai, China). Various proteases (neutral protease, alkaline protease, and trypsin) were incorporated into the blend, which was then subjected to enzymatic hydrolysis at varying temperatures (35–55 °C) over a designated period (2–4 h). After enzymatic hydrolysis, the mixture was boiled to deactivate the enzymes and centrifuged at 2775× *g* at 25 °C for 10 min (H2-16KR, Hunan Kecheng Instrument Co., Changsha, China) to obtain crude fish oil (CO_1_).

The distribution of different mass fractions of ethanol solution and oil in the enzymatic liquid system was explored, and the dotted gizzard shad oil and emulsion layer were separated using a certain mass fraction of ethanol solution. The upper layer of oil was collected, and residual ethanol was removed using a rotary evaporator. The two parts of fish oil were combined to form crude fish oil (CO_2_). The extraction rate and related physicochemical properties of the two types of crude fish oil were then analyzed.

### 2.4. Single-Factor and Response Surface Experimental Design

Experiments focusing on a single factor were carried out to investigate the impact of various elements on the oil extraction rate from dotted gizzard shad, including the type of enzyme, the enzyme dosage, the enzymatic hydrolysis time, the enzymatic hydrolysis temperature, the liquid-to-solid ratio, and the pH level. Based on the results, four main factors with significant effects were selected. A Box–Behnken (BBD) model was then employed to design a four-factor, three-level response surface experiment, with the extraction rate of fish oil serving as the response value.

### 2.5. Fish Oil Refining

The crude fish oil was treated using a one-step degumming and deacidification method. This involved adding a specified amount of ethanol (ranging from 18% to 42% by oil weight) along with NaOH (ranging from 0.28% to 0.56% by oil weight) to a conical flask. The mixture was then shaken for a duration ranging from 15 to 75 min at a controlled temperature between 40 °C and 80 °C. Following the reaction, the mixture was centrifuged at 5180× *g* and 25 °C for 40 min to separate the soap and gums. The optimal conditions, including the amount of NaOH, the duration, the ethanol quantity, and the temperature, were identified based on the outcomes of single-factor experiments. An L9 (3^4^) orthogonal experiment was designed and implemented using SPSS 20.0, referring to the degumming and deacidification method of Bai Dong [22]. Phosphoric acid was used for degumming crude dotted gizzard shad oil to obtain phosphoric acid degummed oil, and NaOH was used to deacidify the phosphoric acid oil to obtain NaOH deacidified oil.

The bleaching and deodorizing steps were adapted and improved from the method of Valéria Terra Crexi et al. [23]. This approach incorporated the use of an adsorbent alongside rotary evaporation, where 5% of the adsorbent, in relation to the oil’s weight, was mixed with the oil and reacted within a rotary evaporator at 60 °C for 30 min. Following this, the mixture was centrifuged at 5180× *g* and 25 °C for 10 min to separate the adsorbent, resulting in refined dotted gizzard shad fish oil. The technique for determining the bleaching rate was improved based on Yang Mengnan et al. [24]. This involved taking a sample from a control group, using petroleum ether as a reference liquid, and conducting optimal absorbance spectral scanning in a UV spectrophotometer (752N, Shanghai NOKO Instruments Co., Shanghai, China) to obtain the maximum absorption wavelength (450 nm). The absorbance of the samples before and after bleaching was measured with petroleum ether as the reference, and the bleaching rate (Y) was calculated using Formula (1):

Y = (A_0_ − A_1_)/A_0_ × 100%, (1)

In Equation (1), Y is the bleaching rate, A_0_ is the absorbance before bleaching, and A_1_ is the absorbance after bleaching.

### 2.6. Physicochemical Properties Determination

The obtained fish oils were characterized according to the national standards of the People’s Republic of China. The phospholipid content was determined by GB 5009.227-2016 [25]; the acid price (AI) was determined by titration (GB 5009.229-2016) [26]; the iodine price (IV) was determined by the specimen’s reaction with Weiss’s reagent, the addition of potassium iodide and water, and the free iodine precipitated by titration with sodium thiosulfate (GB/T 5532-2022) [27]; and the peroxide value (PV) was determined by titration (GB 5009.227-2016) [28], after which the specimen’s reaction with the p-anisidine value (AV) was determined by reacting the specimen with an acetate solution of p-anisidine and then measuring the absorbance at 350 nm.

### 2.7. Fatty Acids Determination

Before analysis, fatty acid methyl esters needed to be prepared. Briefly, about 100 mg of fish oil was dissolved in 5 mL of sodium hydroxide methanol solution (2%, *w*/*v*), and the transesterification reaction was carried out in a water bath at 80 °C ± 1 °C for 60 min. The reaction was stopped by adding 5 mL of boron trifluoride methanol solution (14%, *w*/*v*). A quantity of 10 mL of n-heptane was added with shaking, then 10 mL of saturated sodium chloride solution was added to static layering; the upper layer of n-heptane extract was aspirated; and 3 g of anhydrous sodium sulfate was added with shaking, held static for 5 min on the upper layer of filtrate filtration, and finally added to the injection bottle to be measured. The fatty acid composition was assessed using a gas chromatography system (Agilent 7820A GC; Santa Clara, CA, USA) equipped with an Agilent HP-88 (Agilent HP-88, Santa Clara, CA, USA) column and a split ratio of 30:1. The temperature protocol was set as follows: the injector temperature was set at 270 °C, the detector temperature was set at 280 °C, and the oven temperature was initiated at 100 °C for 1 min. This was followed by an increase at a rate of 15 °C/min to 190 °C, where it was maintained for 4 min. The temperature was then raised at 1 °C/min to 200 °C, held for 15 min, further increased at 1 °C/min to 205 °C, held for 4 min, and finally escalated at 5 °C/min to 240 °C, holding for 9 min, culminating in a total run time of 52 min. Fatty acids were qualitatively identified by comparing them with known standards, and quantification was achieved through the area normalization method. Each sample was analyzed in triplicate to ensure accuracy and reliability of the results.

### 2.8. Volatile Components Determination

The detection of volatile components in fish oil was carried out using a gas chromtographyion mobility spectrometry instrument (Flavorspec, G.A.S.^®^ Instrument, Hanon Advanced Technology Group Co., Ltd., Jinan, China). A 5 g portion of the sample was placed in a 20 mL headspace vial. High-purity nitrogen was employed both as the carrier gas for the gas chromatography process and as the drift gas for ion mobility spectrometry. The analysis was carried out under the following conditions: a detector temperature of 60 °C and an IMS temperature of 45 °C. The sample was incubated at 60 °C for 15 min before 500 μL of it was injected into the system. The needle temperature was set at 85 °C, with the incubation speed set at 500 rpm. The GC conditions were as follows: E1 (drift gas flow rate) at 150 mL/min, E2 (carrier gas flow rate) starting at 2 mL/min, holding for 2 min, then increasing to 10 mL/min over 8 min, followed by an increase to 100 mL/min over 10 min, and holding for 10 min, with each sample analyzed in triplicate. The spectra obtained were both qualitatively and quantitatively analyzed using the GC-IMS library in VOCal.

### 2.9. Key Volatile Components Identification Method

The key flavor compounds during the refining process of dark fish oil were analyzed using the relative odor activity value (ROAV) method, which was integrated with the importance of predictive variables projection (VIP) values, as suggested by Pei et al. [29]. The formula for calculating ROAV is as follows:

(2) $$\mathrm{ROAV}=\frac{\mathrm{CA}}{\mathrm{TA}}\times \frac{\mathrm{Tstan}}{\mathrm{Cstan}}\times 100$$

In Equation (2), CA represents the relative percentage content of compound A; TA represents the aroma threshold of compound A, which can be obtained from the literature [30,31] in mg/kg; and Tstan and Cstan indicate the aroma threshold and the relative percentage content, respectively, of the compound that contributes most significantly to the flavor of the sample.

### 2.10. Statistical Analysis

All experiments were conducted in triplicate, and the data collected from these experiments were processed and analyzed utilizing SPSS version 20.0. The findings were reported as the mean value plus or minus the standard deviation. To evaluate the significance of the differences observed, a two-way analysis of variance (ANOVA) was applied, with a *p*-value of less than 0.05 considered as indicative of significant differences. Additionally, orthogonal partial least squares discriminant analysis (OPLS-DA) was carried out using SIMCA software version 14.1 to determine the importance of predictive variables projection (VIP).

## 3. Results and Discussion

### 3.1. Nutritional Components of Dotted Gizzard Shad

The dotted gizzard shad was found to contain 59.94 ± 0.49% moisture, 16.62 ± 0.45% protein, 22.33 ± 0.04% fat, and 0.64 ± 0.04% ash. Notably, its fat content was considerably higher than that of herring, which has a fat content of 9.0% [9], and far exceeded the fat content in the viscera of deep-sea tuna (5.13%) [22]. Xiao Yuejuan et al. [32] studied the nutritional components of dotted gizzard shad meat and reported that the fat content in dotted gizzard shad meat was only 3.83%, a figure significantly lower than the results of this study, indicating a high fat concentration in the viscera of dotted gizzard shad. These findings underscored the suitability of dotted gizzard shad as an excellent source for extracting fish oil, offering potential for large-scale industrial production.

### 3.2. Optimization of Dotted Gizzard shad Oil Extraction Parameters

#### 3.2.1. Protease Screening

**Figure 1:**
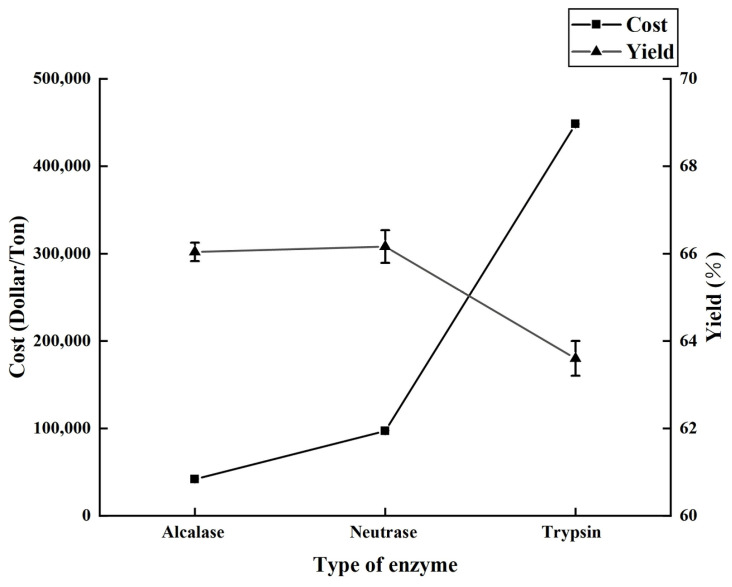
The impact of various enzymes on both the yield of extraction and the cost of production, emphasizing their effects on fish oil products.

Figure 1 illustrates the impact of neutral protease, trypsin, and alkaline protease on both the extraction efficiency and production cost of dotted gizzard shad oil. According to the data presented, when each enzyme was used under its ideal reaction conditions, both alkaline protease and neutral protease led to extraction rates that were higher than those achieved with trypsin. Notably, the use of alkaline protease resulted in a significant reduction in production costs, saving USD 55,293.82 for each ton of oil produced compared with the use of trypsin. This indicates the potential of alkaline protease to enhance hydrocarbon extraction rates at the lowest possible cost. The impact of various enzymes, enzyme concentrations, and extraction times on the rate of extraction was investigated by Ella Aitta et al. [7]. The researchers discovered that using alkaline protease led to the highest oil yield when the enzymatic hydrolysis was conducted for 105 min at an enzyme concentration of 0.4%. Furthermore, the quality of the oil extracted under these conditions was found to be satisfactory.

#### 3.2.2. Single-Factor Experiments for Extracting Dotted Gizzard Shad Oil

**Figure 2:**
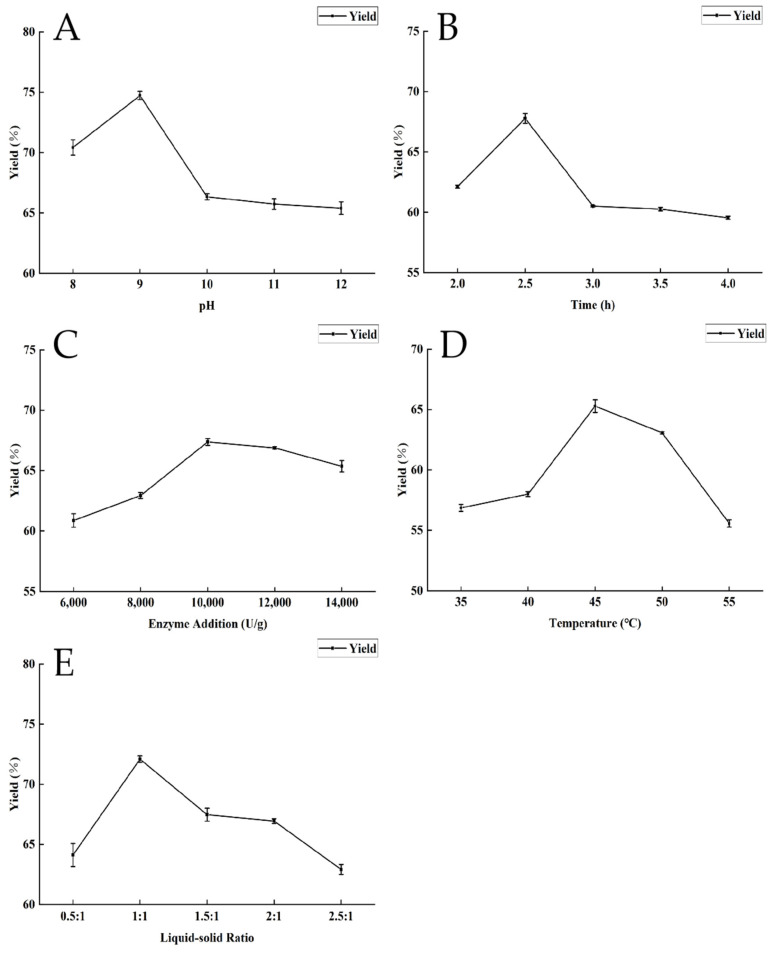
Single-factor experiment on ethanol-assisted enzymatic hydrolysis of dotted gizzard shad oil. (**A**) Influence of pH on the dotted gizzard shad oil extraction efficiency. (**B**) Impact of hydrolysis duration on the oil extraction rate from dotted gizzard shad. (**C**) Effect of enzyme addition on the extraction rate of dotted gizzard shad oil. (**D**) Relationship between hydrolysis temperature and dotted gizzard shad oil extraction rate. (**E**) Effect of liquid–solid ratio on the extraction rate of dotted gizzard shad oil.

Through single-factor experiments, the effects of various parameters, such as enzymatic hydrolysis duration, temperature, liquid-to-solid ratio, pH, and enzyme concentration, on the extraction efficiency of dotted gizzard shad oil were examined, as illustrated in Figure 2. The extraction rate of dotted gizzard shad oil exhibited an initial increase followed by a subsequent decrease as the pH increased, culminating in its peak value at pH 9.0 (Figure 2A). This pattern arose because the pH influenced both the charge characteristics of the substrate and the protease, thereby affecting the stability of the enzyme molecules [22]. Figure 2B shows the impact of enzymatic hydrolysis time on the extraction rate, where the maximum oil extraction rate was achieved at 2.5 h, followed by a gradual decline. This could be attributed to the enhanced interaction between the alkaline protease and the substrate over time until a balance is achieved. Further extending the time could lead to the formation of an emulsion, which hinders the release of oils [33]. Figure 2C demonstrates that the extraction rate was highest at an enzyme concentration of 10,000 U/g. Beyond this point, the addition of more enzymes could hinder hydrolysis due to either the enzymes’ self-digestion or their inhibitory effects. Moreover, because alkaline protease is a protein with amphiphilic properties, it can act as a surfactant and foster emulsion layer formation, negatively impacting the extraction rate of dotted gizzard shad oil [22,34]. The influence of enzymatic hydrolysis temperature on dotted gizzard shad oil, as shown in Figure 2D, indicates that the extraction rate reached its maximum at 45 °C and decreased with further temperature increases. This is likely because a suitable temperature rise enhances the reaction kinetics and equilibrium, but excessively high temperatures may cause protease denaturation and reduce enzyme efficacy, thus decreasing the oil extraction rate [35]. The liquid-to-solid ratio showed a consistent trend with the first four factors, as depicted in Figure 2E, peaking at a 1:1 ratio. This was because adequate water volume ensured thorough interaction between the protease and substrate, enhancing the hydrolysis process and its extent. However, an excessive water volume might dilute the substrate concentration, leading to incomplete hydrolysis and a reduced extraction rate of fish oil [34].

#### 3.2.3. Response Surface Optimization Experiment for Extracting Dotted Gizzard Shad Oil

Based on the results of the single-factor experiments, the enzymatic hydrolysis time (2, 2.5, and 3 h), temperature (40, 45, and 50 °C), liquid-to-solid ratio (0.5:1, 1:1, and 1.5:1), and pH (8, 9, and 10) were selected as the four key factors for the response surface experiment design. The Design Expert 12.0 software, utilizing linear regression and ANOVA, generated a quadratic polynomial regression equation for the extraction rate (%) of dotted gizzard shad oil as follows: 75.4 − 0.53A + 0.39B − 1.31C + 1.46D − 1.20AB − 0.98AC − 1.13AD − 0.95BC − 1.04BD − 1.76CD − 2.53A2 − 2.13B2 − 1.2C2 − 5.64D2. The experimental results are shown in Table S1, and the analysis results are presented in Table 1, where the *p*-value < 0.0001, and R^2^ (0.9917) and Adj R^2^ (0.9835) are both greater than 0.95 and differ by less than 0.2, indicating that the model is highly significant with good fitting and predictive ability. The coefficient of variation (CV) reflects the reproducibility of the model, and previous studies have indicated that a model is considered reproducible when the CV is less than 10% [36]. The CV of 0.61 in Table 1 demonstrates good repeatability. Table 1 highlights the liquid-to-solid ratio as the most influential factor on the oil extraction rate, followed by the enzymatic hydrolysis temperature, pH, and hydrolysis duration. Interestingly, there was a significant interaction between the liquid-to-solid ratio and the pH and between the hydrolysis time and the temperature. The optimal conditions for enzymatic hydrolysis were observed to be pH 9.02, enzymatic hydrolysis time 2.56 h, temperature 43.17 °C, and liquid-to-solid ratio 1.07:1, with a theoretical oil extraction rate of 75.49%. Three parallel verification experiments were performed under these conditions, yielding an oil extraction rate of 74.94 ± 0.45%, which is very close to the theoretical extraction rate. This concordance underscores the ethanol-assisted enzymatic hydrolysis method’s strong reproducibility and operational feasibility. Nahidur Rahman et al. [37] discussed the effect of different extraction methods of pangus fish oil on the extraction rate and found that the microwave-assisted extraction method, the aqueous extraction, and the acidic silage method did not provide more than a 30% extraction rate. Ella Aitta et al. [7] used alcalase, neutrase, and protamex to extract fish oil from sea herring with extraction rates ranging from 37 to 58%. Yu-Hsiang Wang et al. [38] showed that pretreatment of cobia livers with papain resulted in a 38% extraction rate. These findings show that ethanol-assisted enzymatic hydrolysis has a greater extraction rate than other commonly used techniques.

### 3.3. Optimization of Refining Parameters for Dotted Gizzard Shad Oil

#### 3.3.1. Single-Factor Experiments for One-Step Degumming and Deacidification

**Figure 3:**
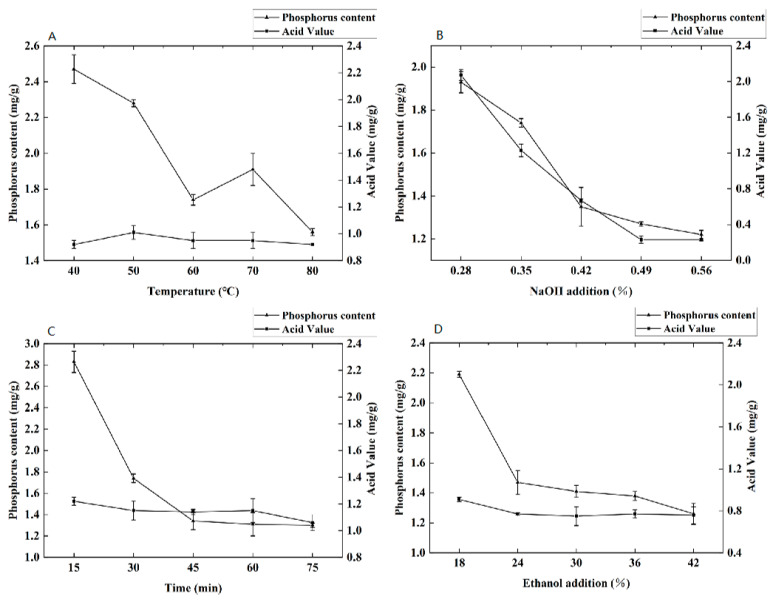
Single-factor experiment on one-step degumming and deacidification refining of dotted gizzard shad oil. (**A**) Impact of temperature on one-step degumming and deacidification refining of dotted gizzard shad oil. (**B**) Influence of NaOH addition on one-step degumming and deacidification refining of dotted gizzard shad oil. (**C**) Effect of time on one-step degumming and deacidification refining of dotted gizzard shad oil. (**D**) Impact of ethanol addition on one-step degumming and deacidification refining of dotted gizzard shad oil.

The temperature is one of the most important factors affecting the effect of degumming and deacidification because higher temperatures can reduce the viscosity of fats and oils, but in order to prevent the oxidation of fats and oils at too high a temperature, the reaction temperature should not exceed 90 °C [16,39]. In this study, temperatures ranging from 40 to 80 °C were tested to determine the ideal conditions for the one-step degumming and deacidification method. As illustrated in Figure 3A, the acid value of dotted gizzard shad oil remained relatively unchanged (<1.0 mg/g) across this temperature spectrum, indicating that the one-step degumming and deacidification method had a good deacidification effect. The phospholipid content decreased from 2.47 ± 0.08 mg/g to 1.74 ± 0.03 mg/g as the temperature increased from 40 °C to 60 °C and slightly increased when the temperature reached 70 °C. This slight rise in phospholipid levels could be attributed to the enhanced evaporation of ethanol at elevated temperatures, which may form a vapor barrier limiting molecular migration into the ethanol phase, slightly elevating the phospholipid levels in the oil [40]. Therefore, the optimal temperature for the one-step degumming and deacidification process is 60 °C.

Figure 3B illustrates the effect of the quantity of NaOH added on both the acid value and phospholipid content. With increasing amounts of NaOH, there was a gradual decrease observed in both the acid value and phospholipid content of the dotted gizzard shad oil. Once the addition of NaOH surpassed 0.49%, there was minimal change noted in the acid value, and the trend in phospholipid alteration began to level off. During this process, NaOH reacts with free fatty acids to produce fatty acid salts and water. The adsorptive properties of the resulting soap facilitate the absorption of some impurities present in the oil. Moreover, as the pH level rises, proteins and other compounds tightly bound to phospholipids start to degrade or dissolve in the aqueous phase, leading to the release of free phospholipids and thereby removing them from the oil [41]. Hence, 0.49% was the optimal NaOH addition amount for the one-step degumming and deacidification process.

Figure 3C illustrates the influence of time on the one-step degumming and deacidification process. The acid value exhibited minimal variation with increasing time, maintaining a value of 1.22 ± 0.03 mg/g at 15 min, with subsequent values remaining below 1.2 mg/g. This observation could be attributed to the swift reaction between NaOH and the free fatty acids present in the dotted gizzard shad oil upon introduction into the reaction system [16]. However, the phospholipid content rapidly decreased as the duration of time was increased but became stable after exceeding 45 min. Considering that the reaction between NaOH and phospholipids required time to achieve equilibrium, a duration of 60 min was determined as the optimal timeframe for the one-step degumming and deacidification process. This duration can significantly shorten the reaction time compared with the traditional degumming and deacidification processes and eliminates washing steps, saving production costs [42].

Figure 3 demonstrates the effect of incorporating ethanol on the degumming and deacidification processes. The pattern observed in the change in acid value parallels those seen in Figure 3A,C, showing no substantial fluctuations in the acid value of dotted gizzard shad oil across five ethanol concentration gradients, all remaining under 1.0 mg/g. After obtaining insights from Figure 3A–D, it becomes clear that the free fatty acid levels in dotted gizzard shad oil were predominantly influenced by NaOH. Moreover, the phospholipid content exhibited a decline as the ethanol concentration increased, with the rate of reduction diminishing after exceeding an addition amount of 24%. This phenomenon could be explained by a moderate increase in the water content within the system, which facilitated the hydration and coalescence of phospholipids, causing their subsequent precipitation and separation from the oil. The diminishing phospholipid content with escalating ethanol concentrations was presumably due to the solvent’s polarity aligning more closely with that of phospholipids, thus enhancing the solubility of phospholipids in the solvent [43,44]. Therefore, the optimal ethanol addition for the one-step degumming and deacidification process was 24%.

#### 3.3.2. Orthogonal Experiment for One-Step Degumming and Deacidification

An orthogonal experiment was conducted to optimize the one-step degumming and deacidification process, building upon the findings of single-factor trials. Interestingly, temperature, time, and ethanol addition demonstrated no significant impact on the acid value in the study. Given the consistently low acid value of the fish oil at the chosen NaOH addition levels, the assessment of degumming and deacidification effects relied on the phospholipid content. The statistical software SPSS 20.0 was used to conduct range analysis, and the results are displayed in Table 2. According to the *p*-values, the ethanol addition amount (B) exerted the most considerable influence on degumming and deacidification, followed by temperature (C) and time (D), with the NaOH addition amount (A) exhibiting the least impact. Consequently, the hierarchy of influence for each factor is B > C > D > A. Based on the k-values, the optimal combination of conditions was determined to be A3B3C2D3, i.e., NaOH addition amount of 0.56%, ethanol addition amount of 30%, temperature at 60 °C, and time at 60 min. Under these process conditions, the degumming and deacidification effects were found to be the maximum.

### 3.4. Physicochemical Properties of Dotted Gizzard Shad Oil

Table 3 presents the acid value (AV), iodine value (IV), peroxide value (PV), anisidine value (p-AV), and extraction rate, at different stages of the refining process of dotted gizzard shad oil, showing significant differences in the physicochemical properties at each stage (*p* < 0.05). Generally speaking, the physicochemical properties of dotted gizzard shad oil before and after refining were in accordance with the standards of the aquatic industry of the People’s Republic of China (SC/T3502-2016) [45]. AI serves as an indicator of the free fatty acid content in fish oil, with elevated levels of free fatty acids potentially hastening oil oxidation and resulting in undesirable odors. Although free fatty acids are present in minimal quantities in living tissue lipids, they are liberated from lipids following enzymatic or thermal treatment, necessitating control of the acid value postenzymatic hydrolysis [46]. As seen in Table 3, the acid value of crude dotted gizzard shad oil significantly decreased after degumming and deacidification, mainly because the added NaOH could effectively neutralize the free fatty acids in the dotted gizzard shad oil. Additionally, the acid value of fish oil tended to increase after the deacidification treatment, owing to acid addition in traditional deacidification processes [23]. In contrast, the one-step degumming and deacidification processes demonstrated a more substantial efficacy in eliminating free fatty acids. Furthermore, the use of activated bleaching earth in bleaching and deodorization exhibited a deacidifying effect, as observed by Chakraborty et al. [47].

IV is proportional to the number of double bonds present in the fatty acids of fish oil, representing the degree of unsaturation. Thus, it is directly proportional to the degree of unsaturation [48]. As depicted in Table 3, the iodine value of fish oil at each stage generally exhibited an upward trend, ranging from 127.96 ± 1.13 g/100 g to 145.78 ± 0.13 g/100 g. This trend suggests an increase in the degree of unsaturation in the refined dotted gizzard shad oil, indicating the effective removal of impurities during the refining process.

PV mainly primarily reflects the presence of hydroperoxides and serves as a crucial indicator for assessing oil quality and the degree of oxidation [49]. As shown in Table 3, there was no significant reduction in the peroxide value when comparing degummed and deacidified fish oil with crude fish oil (*p* > 0.05). This observation could be attributed to the relatively low peroxide value of the crude fish oil (2.99 ± 0.02 meq/kg), indicating a limited presence of oxidation products. Consequently, their removal through hydration gel adsorption alone may not be pronounced [50]. Following treatment with activated bleaching earth adsorption and simultaneous rotary evaporation, the peroxide value drops dramatically because activated bleaching earth can adsorb primary and secondary oxidation products or comparable chemicals [47].

The p-AV serves as an indicator of the formation of non-volatile secondary products resulting from further oxidation and degradation of lipids initiated by hydroperoxide-induced free radical oxidation [51]. In Table 3, there was a significant difference (*p* < 0.05) observed between the degummed and deacidified fish oil and the refined fish oil in terms of p-AV, with the refined fish oil showing a significantly reduced p-AV. This result is contrary to the findings of Zhang Quan [50], potentially because in their study, the adsorption by activated bleaching earth and rotary evaporation were conducted separately, whereas in this experiment, deodorization and bleaching were carried out under a vacuum of −0.1 MPa with activated bleaching earth adsorption. The combined action of rotary evaporation and activated bleaching earth can effectively reduce the possibility of lipid contact with oxygen during the reaction process, and the activated bleaching earth, serving as a solid-phase adsorbent, can eliminate the oxidation of various products, consistent with the results reported by Song et al. [52].

The results showed that the use of 40% ethanol solution could effectively separate the spotted gizzard shad oil from the emulsified layer and improve the fish oil extraction rate. There was no significant difference in acid value between CO_1_ and CO_2_, with only a slight increase in peroxide value. This slight increase may be attributed to the small amount of hydroperoxides produced by the heat of the fish oil during the treatment of the ethanol–fish oil mixture by rotary evaporation. Furthermore, the ethanol-assisted enzymatic hydrolysis method exhibited a significantly higher extraction rate (8.31 ± 0.83%) compared with both the post-steaming pressing method and the 40% ethanol extraction method (2.81 ± 0.28%) [53]. The notable increase in extraction rate, along with the non-toxicity and recoverability of the ethanol solution, underscored the superiority of the ethanol-assisted enzymatic method. This method not only reduced production costs but also opened up new possibilities for fish oil extraction processes. When compared with traditional fish oil obtained after degumming with phosphoric acid and deacidifying with NaOH, the one-step degummed and deacidified fish oil exhibited lower acid and peroxide values, indicating superior product quality. Additionally, the production process generated no wastewater and operated at lower temperatures.

### 3.5. Fatty Acid Composition

Table 4 illustrates that both crude and refined dotted gizzard shad oil exhibited identical compositions, comprising 22 fatty acids ranging from C12 to C24. This included eight saturated fatty acids (SFAs), six monounsaturated fatty acids (MUFAs), and eight polyunsaturated fatty acids (PUFAs). In both crude and refined fish oil, SFAs account for 38.51% and 38.23%, MUFAs for 37.21% and 37.86%, and PUFAs for 24.28% and 24.92% of the total fatty acids. There was no significant change in fatty acid composition before and after refining, a result that is in accordance with the findings of Valéria Terra Crexi et al. [23]. This preservation is comparable to that observed in fish oils derived from sources such as tuna and mackerel, in terms of the proportions of SFAs, MUFAs, and PUFAs [14,54].

Among SFAs, palmitic acid (C16:0) has the highest proportion in both crude and refined fish oil, accounting for 65.85% and 65.66% of total SFAs, followed by myristic acid (C14:0) and stearic acid (C18:0). In MUFAs, oleic acid (C18:1) is the most abundant in both crude and refined fish oil, followed by palmitoleic acid (C16:1), together constituting 91.88% and 91.86% of the total MUFAs in crude and refined fish oil, respectively. Studies have shown that palmitoleic acid and oleic acid can regulate cardiovascular metabolism and reduce the risk of type II diabetes [55,56]. Among PUFAs, EPA and DHA contents are similar, together accounting for 82.45% and 82.07% of the total PUFAs in crude and refined fish oil. EPA and DHA play pivotal roles in fetal development and promote healthy aging in adults, with DHA particularly recognized as a fundamental component of cell membranes, notably abundant in the brain and retina [57].

In summary, the refined dotted gizzard shad oil exhibits a higher proportion of total UFAs in its fatty acid composition, particularly with an increased content of PUFAs, which are recognized as a nutritional benchmark for fish oil. Moreover, traditional methods of fish oil refinement often result in oxidative degradation of PUFAs due to prolonged exposure to high temperatures, leading to the loss of nutritional value and a decline in oil quality [58]. The refining process proposed in this study, with a maximum temperature not exceeding 60 °C, addressed this concern by eliminating the drawbacks associated with lipid oxidation and degradation under high temperatures in traditional processes. Consequently, this method effectively preserved the nutritional components of dotted gizzard shad oil to the fullest extent.

### 3.6. Volatile Compounds

#### 3.6.1. Volatile Components and Their Relative Contents

**Figure 4:**
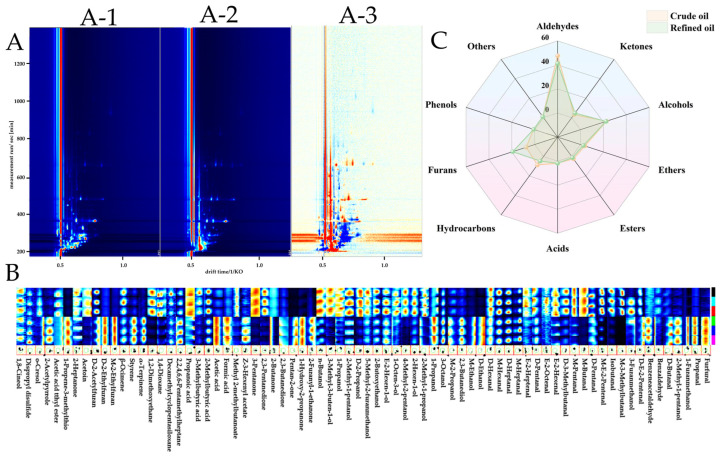
Gas chromatography-ion mobility spectrometry (GC-IMS) spectrogram of volatile compounds. (**A**(**A-1**–**A-3**)): Two-dimensional spectra of refined oil and fish oil. (**B**) Fingerprint spectra of refined oil and crude oil. (**C**) Radar chart of relative content of volatile components in refined oil and crude oil.

GC-IMS was employed to examine the volatile components in both crude and refined dotted gizzard shad oil. The two-dimensional chromatograms of GC-IMS are illustrated in Figure 4A, with the morphology chart of crude fish oil serving as a reference. The horizontal axis denotes the ion mobility time, while the vertical axis represents the retention time of gas chromatography. The red vertical line signifies the reaction ion peak (RIP), and the signal intensity is depicted by the color [59]. Figure 4(A-1–A-3) distinctly reveal notable discrepancies between the two chromatograms, with more blue areas evident in the comparison chart. This indicates a reduction in the content of certain substances in the refined fish oil in comparison with the crude oil, showcasing significant refining effects. Additionally, a fingerprint chromatogram was utilized to compare the content of each volatile substance before and after the refining of the dotted gizzard shad oil, as depicted in Figure 4C. Each column in the figure represents a compound, with aldehydes and alcohols exhibiting a noteworthy reduction in signal strength. This observation aligns with the relative content changes of these two types of compounds displayed in Figure 4B. A total of 73 kinds of volatile substances were detected in both crude and refined fish oil, including 20 aldehydes, 20 alcohols, 4 ethers, 3 esters, 8 ketones, 4 hydrocarbons, 4 furans, 1 phenol, 5 acids, and 4 others. Although the types of volatile compounds remained the same before and after refining, differences existed in their relative contents, as shown in Figure 4B. Overall, after refining, there was a significant decrease in the relative content of aldehydes and hydrocarbons in dotted gizzard shad crude fish oil. Additionally, the relative contents of alcohols, ethers, esters, phenols, and other categories exhibited minor reductions, while the relative content of furans experienced a significant increase. Moreover, there were slight increases in the relative contents of alcohols and acids. Aldehydes have emerged as the most prevalent compounds in both crude and refined dotted gizzard shad oil, serving as primary products of lipid secondary oxidation. With their low sensory thresholds, aldehydes exert a significant impact on the flavor and quality of fish oil [60]. Table 5 indicates that aldehydes mainly include hexanal (D,M), (E)-2-pentenal (D,M), propanal, pentanal (D,M), and 3-methylbutanal (D,M), among 20 types of compounds. With the exception of propanal, furfural, and butanal (D,M), the relative concentrations of the remaining aldehyde compounds decrease to varying degrees after refining, resulting in an overall reduction in the relative content of aldehyde compounds. Propanal, a secondary oxidation product of omega-3 fatty acids [61], may increase during refining due to activated bleaching earth in the deodorization and bleaching stages catalyzing the degradation of primary oxidation products of dotted gizzard shad oil into a large amount of aldehydes [50]. Additionally, the slight decrease in EPA + DHA content in refined fish oil compared with crude fish oil might also be related to this result. Alkanals such as hexanal, pentanal, butanal, and heptanal, reported in many studies on oil volatility, are considered important volatile flavor components [62,63,64] and represent the oxidation level of fats to some extent. Their decrease in Table 5 suggests significant refining effects. The aldehydes derived from oleic acid, such as butanal (D,M) and hexanal (D,M), can impart grassy and fruity aromas to fish oil [62,65], along with small amounts of aromatic aldehydes like benzaldehyde and benzene acetaldehyde, which have an almond aroma.

Alcohols are generally produced by the reduction of secondary hydroperoxides or carbonyl compounds of fatty acids [66], and they are categorized into saturated and unsaturated alcohols. Saturated alcohols generally possess higher odor thresholds compared with unsaturated alcohols [67]. However, alcohols, in general, have high odor thresholds and usually do not exert a significant influence on the flavor of fats [68]. The relative content changes in alcohols were not significant, but 2-propanol (M) exhibited the highest relative content in crude fish oil and significantly increased after refining, possessing strong antibacterial capabilities [69], potentially extending the shelf life of fish oil products. Oct-1-en-3-ol is a prominent unsaturated alcohol that may contribute a grassy and mushroom-like flavor to fish oil. However, its production primarily occurs through the oxidation of arachidonic acid and linoleic acid [70], and its levels tend to increase with prolonged storage time, which could compromise the preservation of fish oil.

Ketones and acids are both derived from the oxidative decomposition of lipids [30]. Among these, crude fish oil contains minimal amounts of 2-heptanone [71], which serves as an indicator of product spoilage and diminishes even further after refining. Acidic substances, known for their sweaty, irritating, and unpleasant odors [72], have very high odor thresholds. Therefore, even if the content of acidic substances in refined fish oil increases, it is unlikely to have a significant impact on the flavor of the fish oil. The relative contents of esters and ethers, which constitute a small proportion in fish oil, decreased further after refining. All substances in the ester category decreased in their relative contents, possibly due to the degumming process [50]. Additionally, the substance 1-propene-3-methylthio, formed during the degradation of allicin [73], is not a component of fish oil products and is presumed to be a contaminant from the extraction head coating or chromatographic column materials. Hence, it is not subjected to detailed analysis here. Hydrocarbons typically do not significantly influence the flavor of fish oil due to their high odor sensitivities. However, certain hydrocarbons may enhance the overall flavor profile or, in some instances, generate ketones and aldehydes, which are considered potential contributors to flavor [74]. Moreover, furans, pyrroles, and monoterpenes have been identified in dotted gizzard shad oil, with the relative concentration of furans observed to increase in refined fish oil. Furans, characterized by their low odor thresholds, can exert a notable impact even at low concentrations, imparting sweet and caramel-like notes to fish oil [75].

#### 3.6.2. Analysis of Differences in Volatile Components

**Figure 5:**
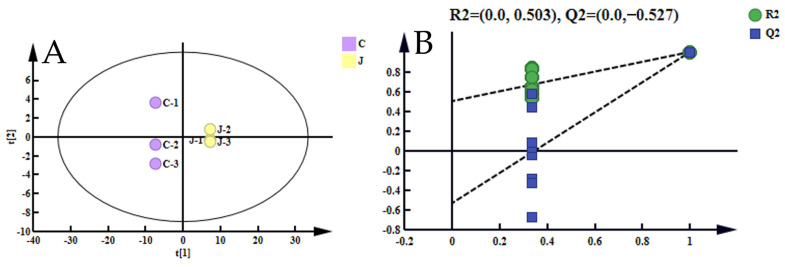
(**A**) OPLS-DA. (**B**) Permutation test of permutation test of OPLS-DA model.

Orthogonal partial least squares discriminant analysis (OPLS-DA) enabled the differentiation between two sets of data under supervised conditions, facilitating the construction of intergroup difference maps with greater precision [69]. The results, depicted in Figure 5A, revealed significant disparities in volatile substances before and after refining, with a high level of reproducibility within each group. The model’s explanatory power for the independent variables (R^2^X) was measured at 0.945, while for the dependent variables (R^2^Y) it reached 0.999, indicating accuracies of 94.5% and 99.99% on the X and Y axes, respectively. Moreover, the predictive power of the model (Q^2^) was calculated at 0.999, suggesting a recognition rate of 99.99%. Generally, R^2^ and Q^2^ values exceeding 0.5 were considered acceptable for model results, ideally approaching 1 [76]. A permutation test conducted 500 times, as depicted in Figure 5B, revealed that the Q^2^ regression line intersected below 0 on the vertical axis. This observation indicated that the model was not afflicted by overfitting [77].

Furthermore, the VIP value was used to identify key differential substances, with substances having VIP > 1 significantly affecting changes in the volatile components of fish oil. Based on this criterion, 59 differential substances between crude and refined fish oil were selected. The overall flavor of fish oil was determined by the aroma characteristics of volatile substances, their relative content, and their thresholds [78]. Thus, the 59 differential substances were subjected to analysis using the relative odor activity value (ROAV) to pinpoint key flavor compounds, as delineated in Table 6. Compounds with higher ROAV values were considered to contribute more significantly to the overall flavor of the sample, with ROAV ≥ 1 serving as the criterion for evaluating key volatile compounds [79]. In this study, 19 key volatile compounds were identified, including hexanal (D,M), 3-methylbutanal (D), isobutanal, pentanal (M), oct-1-en-3-ol, and methyl 2-methylbutanoate with higher ROAVs, contributing significantly to the aroma of dotted gizzard shad oil, imparting grassy, fruity, earthy, nutty, and mushroom flavors. Notably, among these key volatile compounds, except for heptanal (D,M), hexanal (D,M), propanal, butanal (D), and benzene acetaldehyde, the ROAVs of other substances decreased to varying degrees, especially oct-1-en-3-ol, which has been reported as a major source of earthy–musty odors in aquatic products [80]. Its ROAV decrease after refining indicated that refining effectively removed some volatile substances with undesirable flavors from fish oil.

## 4. Conclusions

This study determined the optimal process for ethanol-assisted enzymatic hydrolysis, a novel method for extracting fish oil, as follows: pH 9.02, enzyme digestion time 2.56 h, enzyme digestion temperature 43.17 °C, and liquid-to-solid ratio 1.07:1. These parameters yielded high-quality crude fish oil from dotted gizzard shad, presenting a sustainable, environmentally friendly, and high-yield extraction approach. Furthermore, the optimal process for ethanol-NaOH one-step degumming and deacidification method was established: NaOH addition of 0.56%, ethanol addition of 30%, temperature of 60 °C, and a duration of 60 min. This method addressed the challenges of traditional degumming and deacidification processes, such as cumbersome procedures, high reaction temperatures, and potential wastewater generation. During the refining process, significant improvements in the quality of dotted gizzard shad oil were observed (*p* < 0.05). Notably, there were no significant changes in the fatty acid composition (*p* > 0.05), while the relative content of volatile substances showed significant alterations (*p* < 0.05). These findings suggest that the extraction and refining process proposed in this study could serve as an alternative to traditional fish oil preparation methods. Moreover, utilizing the inexpensive, abundant, and nutritious dotted gizzard shad as a new source of fish oil presents a promising opportunity in the field of nutritional science and food technology.

## Figures and Tables

**Table 1:** Analysis of variance (ANOVA) for the response surface quadratic model.

Source	Sum of Squares	df	Mean Square	F Value	*p*-Value
Model	311.77	14	22.27	119.98	<0.0001
A	3.40	1	3.40	18.33	0.0008
B	1.83	1	1.83	9.83	0.0073
C	20.57	1	20.57	110.81	<0.0001
D	25.46	1	25.46	137.18	<0.0001
AB	5.78	1	5.78	31.16	<0.0001
AC	3.84	1	3.84	20.70	0.0005
AD	5.11	1	5.11	27.52	0.0001
BC	3.63	1	3.63	19.55	0.0006
BD	4.28	1	4.28	23.09	0.0003
CD	12.32	1	12.32	66.38	<0.0001
A^2^	41.40	1	41.40	223.03	<0.0001
B^2^	29.36	1	29.36	158.18	<0.0001
C^2^	9.32	1	9.32	50.22	<0.0001
D^2^	206.15	1	206.15	1110.67	<0.0001
Residual	2.60	14	0.19		
Lack of fit	2.42	10	0.24	5.31	0.0609
Pure error	0.18	4	0.046		
Cor total	314.37	28			
R^2^	0.9917				
Adj R^2^	0.9835				
CV%				0.61	

**Table 2:** Analysis of L_9_(3)^4^ test results.

NO.	A-NaOH Addition	B-Ethanol Addition	C-Temperature	D-Time	Phosphorus Content
1	0.56	24	70	30	2.32
2	0.56	30	50	45	1.93
3	0.49	18	70	45	3.08
4	0.49	30	60	30	1.47
5	0.49	24	50	60	1.85
6	0.42	30	70	60	2.06
7	0.42	18	50	30	3.51
8	0.56	18	60	60	1.86
9	0.42	24	60	45	1.92
k1	2.497	2.796	2.409	2.412	
k2	2.134	2.031	1.753	2.312	
k3	2.039	1.843	2.486	1.946	
R	0.458	0.952	0.754	0.467	

**Table 3:** Quality properties of dotted gizzard shad at different stages.

Process Stage		Acid Value (mg/g)	Iodine Value (mg/g)	Peroxide Value (meq/kg)	Anisidine Value	Extraction Rate (%)	Bleaching Rate (%)	Phospholipid Content (mg/g)
Crude fish oil (CO_1_)		3.20 ± 0.09 ^b^	127.96 ± 1.13 ^b^	2.99 ± 0.02 ^b^	-	74.94 ± 0.45% ^a^	-	-
Crude fish oil (CO_2_)		3.31 ± 0.01 ^b^	-	3.54 ± 0.06 ^a^	-	58.93 ± 0.50% ^b^	-	6.80 ± 0.01
Degummed with phosphoric acid		4.93 ± 0.5 ^a^	-	2.42 ± 0.1 ^c^	-	-	-	-
Deacidified with NaOH		0.99 ± 0.07 ^c^	-	1.11 ± 0.03 ^d^	-	-	-	-
One-step degumming and deacidification		0.31 ± 0.01 ^b^	142.92 ± 1.15 ^a^	2.89 ± 0.10 ^b^	14.45 ± 0.69 ^a^	-	-	1.18 ± 0.01
Deodorized		0.23 ± 0.02 ^c^	145.78 ± 0.13 ^a^	0.42 ± 0.09 ^e^	6.25 ± 0.24 ^b^	-	88.04 ± 0.02%	-
SC/T3502-2016 ^1^	Crude oil	≤8.0	≥120	≤12	-	-	-	-
	Refined oil	≤1.0	≥140	≤5.0	≤20.0	-	-	-

^a–e^ The presence of different superscript letters on the same line indicates significant differences (*p* < 0.05). ^1^ SC/T3502-2016: Fish oil of the People’s Republic of China aquatic industry standards.

**Table 4:** Fatty acid composition of crude oil and refined oil.

Fatty Acid Composition	Crude Oil	Refined Oil
C12:0	0.1 ± 0.00	0.09 ± 0.00
C14:0	8.32 ± 0.01	8.19 ± 0.01
C15:0	0.56 ± 0.00	0.56 ± 0.00
C16:0	25.36 ± 0.01	25.10 ± 0.02
C17:0	0.29 ± 0.00	0.30 ± 0.00
C18:0	3.33 ± 0.00	3.40 ± 0.00
C20:0	0.43 ± 0.01	0.47 ± 0.00
C24:0	0.10 ± 0.01	0.11 ± 0.00
∑SFA	38.51 ± 0.02	38.23 ± 0.04
C14:1	0.11 ± 0.00	0.11 ± 0.00
C16:1	10.82 ± 0.00	10.74 ± 0.00
C17:1	1.58 ± 0.05	1.59 ± 0.00
C18:1	23.37 ± 0.04	24.04 ± 0.06
C20:1	1.20 ± 0.00	1.24 ± 0.01
C24:1	0.12 ± 0.00	0.14 ± 0.00
∑MUFA	37.21 ± 0.02	37.86 ± 0.07
C18:2	1.12 ± 0.01	1.13 ± 0.00
C18:3 n-3	0.28 ± 0.00	0.28 ± 0.00
C18:3 n-6	1.41 ± 0.01	1.47 ± 0.01
C20:2	0.24 ± 0.00	0.26 ± 0.00
C20:3	0.16 ± 0.00	0.16 ± 0.00
C20:4	1.04 ± 0.00	0.99 ± 0.00
EPA	10.92 ± 0.01	10.83 ± 0.01
DHA	9.10 ± 0.01	8.80 ± 0.02
∑EPA + DHA	20.02 ± 0.00	19.63 ± 0.00
∑PUFA	24.28 ± 0.02	23.92 ± 0.04
∑UFA	61.49 ± 0.02	61.78 ± 0.04

**Table 5:** Volatile components of crude oil and refined oil.

	Aldehydes	CAS	RI	RT	DT	Crude Oil	Refined Oil
1	E-2-Heptenal	C18829555	960.7	506.767	1.25659	0.20 ± 0.02%	0.10 ± 0.00%
2	D-Heptanal	C111717	898.9	403.928	1.69466	0.86 ± 0.02%	1.12 ± 0.03%
3	M-Heptanal	C111717	899.2	404.32	1.32824	2.28 ± 0.04%	1.97 ± 0.07%
4	Benzaldehyde	C100527	960	505.461	1.15374	0.18 ± 0.03%	0.18 ± 0.03%
5	E-2-Hexenal	C6728263	799.7	288.287	1.18608	0.98 ± 0.02%	0.34 ± 0.01%
6	D-Hexanal	C66251	788.1	277.246	1.56101	6.42 ± 0.13%	4.14 ± 0.09%
7	M-Hexanal	C66251	791	279.909	1.2547	2.39 ± 0.04%	1.64 ± 0.07%
8	D-E-2-Pentenal	C1576870	746.9	238.597	1.35999	4.17 ± 0.06%	4.04 ± 0.08%
9	M-E-2-Pentenal	C1576870	751	242.189	1.10872	3.79 ± 0.05%	0.75 ± 0.01%
10	Propanal	C123386	812.9	301.49	1.05233	7.90 ± 0.3%	20.62 ± 0.18%
11	Isobutanal	C78842	517	129.568	1.27545	2.15 ± 0.04%	0.36 ± 0.01%
12	Benzeneacetaldehyde	C122781	993.4	571.56	1.25387	0.07 ± 0.01%	0.12 ± 0.01%
13	Furfural	C98011	807	295.499	1.32662	0.03 ± 0.00%	0.09 ± 0.00%
14	D-Pentanal	C110623	693.6	196.123	1.42641	0.98 ± 0.02%	0.63 ± 0.02%
15	M-Pentanal	C110623	689.2	193.011	1.18405	3.02 ± 0.03%	1.37 ± 0.01%
16	D-3-Methylbutanal	C590863	653.2	177.466	1.40501	7.93 ± 0.30%	0.28 ± 0.02%
17	M-3-Methylbutanal	C590863	602	157.669	1.39959	1.25 ± 0.02%	0.39 ± 0.00%
18	E-2-Octenal	C2548870	1065.8	696.914	1.33599	0.08 ± 0.00%	0.04 ± 0.01%
19	D-Butanal	C123728	579.6	149.732	1.28537	2.20 ± 0.03%	3.03 ± 0.01%
20	M-Butanal	C123728	565.4	144.899	1.11597	0.85 ± 0.01%	0.10 ± 0.01%
	**Alcohols**						
21	D-2-Propanol	C67630	511.3	127.881	1.23074	2.12 ± 0.03%	0.41 ± 0.01%
22	M-2-Propanol	C67630	504.8	125.98	1.17873	6.96 ± 0.13%	8.89 ± 0.17%
23	3-Octanol	C589980	1012.5	603.52	1.40111	1.16 ± 0.03%	1.41 ± 0.08%
24	1-Octen-3-ol	C3391864	980.5	545.027	1.15571	0.78 ± 0.11%	0.44 ± 0.01%
25	5-Methyl-2-furanmethanol	C3857258	947.8	483.267	1.26573	0.29 ± 0.02%	0.14 ± 0.01%
26	2-Hexen-1-ol	C2305217	847.9	339.337	1.18191	1.37 ± 0.03%	0.50 ± 0.03%
27	E-2-Hexen-1-ol	C928950	847	338.223	1.51255	0.11 ± 0.00%	0.08 ± 0.01%
28	2-Methylbutanol	C137326	786.9	276.149	1.47139	1.40 ± 0.09%	0.67 ± 0.02%
29	2,3-Butanediol	C513859	793.4	282.217	1.35508	0.91 ± 0.05%	2.25 ± 0.13%
30	4-Methyl-2-pentanol	C108112	773.4	262.954	1.27752	0.39 ± 0.00%	0.08 ± 0.01%
31	3-Methyl-3-buten-1-ol	C763326	738.4	231.198	1.29392	0.19 ± 0.01%	0.04 ± 0.00%
32	2-Methyl-1-propanol	C78831	637.3	171.081	1.17234	2.55 ± 0.02%	0.75 ± 0.03%
33	1-Propanol	C71238	571.6	146.985	1.21978	0.97 ± 0.04%	1.02 ± 0.03%
34	Pentan-1-ol	C71410	764.3	254.299	1.24834	0.51 ± 0.02%	0.24 ± 0.02%
35	D-Ethanol	C64175	447.6	110.388	1.1307	0.21 ± 0.01%	1.72 ± 0.03%
36	M-Ethanol	C64175	466.7	115.384	1.12427	0.24 ± 0.03%	0.96 ± 0.02%
37	3-Furanmethanol	C4412913	975.1	534.352	1.10663	0.33 ± 0.17%	0.21 ± 0.05%
38	n-Butanol	C71363	694.4	196.702	1.38834	1.98 ± 0.07%	0.71 ± 0.01%
39	2-Methyl-1-pentanol	C105306	847.1	338.421	1.29448	0.08 ± 0.00%	0.24 ± 0.02%
40	2-Furanmethanol	C98000	817	305.649	1.12721	0.45 ± 0.01%	1.61 ± 0.04%
	**Ethers**						
41	Dipropyldisulfide	C629196	1107.8	780.639	1.47993	0.57 ± 0.04%	0.53 ± 0.02%
42	2-Butoxyethanol	C111762	903.3	410.397	1.19809	0.40 ± 0.00%	0.21 ± 0.01%
43	1,2-Dimethoxyethane	C110714	645.2	174.204	1.31184	2.66 ± 0.07%	0.29 ± 0.01%
44	1-Propene-3-methylthio	C10152768	700.5	201.214	1.04482	0.22 ± 0.01%	1.50 ± 0.02%
	**Esters**						
45	Methyl 2-methylbutanoate	C868575	774.7	264.229	1.1792	1.69 ± 0.01%	0.68 ± 0.01%
46	Aceticacidethylester	C141786	604.7	158.661	1.10089	0.54 ± 0.01%	0.28 ± 0.01%
47	Z-3-Hexenylacetate	C3681718	1012.7	603.827	1.82125	0.07 ± 0.01%	0.12 ± 0.01%
	**Ketones**						
48	2-Heptanone	C110430	891.1	392.535	1.2576	0.55 ± 0.07%	0.32 ± 0.00%
49	2,3-Pentanedione	C600146	698.5	199.71	1.22401	1.29 ± 0.03%	0.29 ± 0.03%
50	2,3-Butanedione	C431038	568	145.764	1.17656	0.23 ± 0.02%	0.23 ± 0.01%
51	Acetoin	C513860	750.7	241.962	1.32257	0.34 ± 0.01%	0.10 ± 0.01%
52	Pentan-2-one	C107879	677.6	187.761	1.12236	0.09 ± 0.01%	1.05 ± 0.04%
53	3-Pentanone	C96220	647.6	175.171	1.34788	0.18 ± 0.01%	0.05 ± 0.00%
54	2-Butanone	C78933	573	147.466	1.0589	0.12 ± 0.01%	0.10 ± 0.00%
55	1-Hydroxy-2-propanone	C116096	668.6	183.888	1.03674	0.37 ± 0.01%	2.52 ± 0.04%
	**Hydrocarbons**						
56	Decamethylcyclopentasiloxane	C541026	1162.6	905.275	1.82155	1.05 ± 0.08%	0.79 ± 0.09%
57	2,2,4,6,6-Pentamethylheptane	C13475826	896.5	400.362	1.14775	0.68 ± 0.03%	1.05 ± 0.03%
58	1,4-Dioxane	C123911	689.9	193.495	1.32961	6.63 ± 0.04%	1.97 ± 0.02%
59	Styrene	C100425	104.2	898.9	403.829	0.28 ± 0.01%	0.78 ± 0.03
	**Furan**						
60	D-2-Acetylfuran	C1192627	872.1	368.147	1.11778	1.42 ± 0.03%	1.25 ± 0.04%
61	M-2-Acetylfuran	C1192627	909.8	420.436	1.11715	0.17 ± 0.03%	0.65 ± 0.03%
62	D-2-Ethylfuran	C3208160	725.2	220.263	1.04892	4.77 ± 0.21%	13.61 ± 0.33%
63	M-2-Ethylfuran	C3208160	923.7	442.406	1.04686	1.07 ± 0.09%	3.74 ± 0.03%
	**Phenols**						
64	o-Cresol	C95487	988.4	561.097	1.10663	1.02 ± 0.15%	0.93 ± 0.07%
	**Acids**						
65	3-Methylvaleric acid	C105431	934.6	460.515	1.26877	0.42 ± 006%	0.15 ± 0.02%
66	2-Methylbutyric acid	C116530	829.1	318.403	1.20936	0.73 ± 0.01%	0.49 ± 0.01%
67	Acetic acid	C64197	620.1	164.412	1.04808	0.38 ± 0.02%	0.91 ± 0.01%
68	Formic acid	C64186	544.7	138.12	1.04707	0.06 ± 0.00%	0.12 ± 0.00%
69	Propanoic acid	C79094	685.9	191.38	1.26274	0.13 ± 0.03%	0.14 ± 0.01%
	**Others**						
70	β-Ocimene	C13877913	1010	599.523	1.67401	0.72 ± 0.13%	0.46 ± 0.05%
71	α-Terpinene	C99865	1010	599.523	1.22638	0.16 ± 0.03%	0.05 ± 0.00%
72	1,8-Cineol	C470826	1038.3	647.172	1.2951	0.08 ± 0.01%	0.06 ± 0.00%
73	2-Acetylpyrrole	C1072839	1013.1	604.442	1.49339	0.15 ± 0.01%	0.44 ± 0.04%

Note: D- is dimer, and M- is monomer.

**Table 6:** Odor contribution of volatile components of crude oil and refined oil.

Key Compounds	ROAV			Sensory Description
	VIP Score	Crude Oil	Refined Oil	
D-heptanal	1.04682	0.80	1.62	Citrus
M-heptanal	1.01792	2.13	2.85	Citrus
E-2-hexenal	1.06407	1.52	0.81	Grassy
D-hexanal	1.06037	100.00	100.00	Grassy, fatty
M-hexanal	1.05614	37.24	39.63	Grassy, fatty
M-(E)-2-pentenal	1.06447	0.12	0.04	Grassy
Propanal	1.06407	5.43	21.97	Earthy, nutty
D-3-methylbutanal	1.06477	34.30	1.88	Fruity, apple
M-3-methylbutanal	1.06425	5.41	2.62	Fruity, apple
Isobutanal	1.06462	29.54	7.67	-
benzene acetaldehyde	1.00532	0.82	2.17	Fruity, floral
D-pentanal	1.06061	3.82	3.80	Fruity
M-pentanal	1.06382	11.76	8.27	Fruity
(E)-2-octenal	1.04072	1.25	0.97	Fruity, fatty
D-Butanal	1.06353	0.51	1.10	Fruity
M-Butanal	1.06452	0.20	0.04	Fruity
oct-1-en-3-ol	1.00079	36.44	31.88	Mushroom, fatty
2-methyl-1-propanol	1.06446	0.74	0.34	-
methyl 2-methylbutanoate	1.0646	87.72	54.75	Fruity
acetic acid ethyl ester	1.06314	5.05	4.06	Fruity
Acetoin	1.06175	0.29	0.13	Buttery, creamy
Styrene	1.06164	0.26	1.13	Sweet
3-Methyl valeric acid	1.02516	1.96	1.09	Grassy

Note: Substances whose threshold values could not be found are not displayed.

## Data Availability

The original contributions presented in the study are included in the article/Supplementary Material, further inquiries can be directed to the corresponding author.

## Supplementary Materials

The following supporting information can be downloaded at https://www.mdpi.com/article/10.3390/foods13081278/s1: Table S1: Experimental values for the lipid yield by the Box-Behnken design.
