# Supplementary material for: Optimization of Extraction and Refining Parameters of Oil from Dotted Gizzard Shad (Konosirus punctatus)
Source: Foods. 2024 Apr 22;13(8):1278. doi: 10.3390/foods13081278 (PMC11049165; doi:10.3390/foods13081278)
Supplement: Supplementary file 1 [file foods-13-01278-s001.zip › foods-2930947-supplementary.pdf]

Table S1. Experimental values for the lipid yield by the Box-Behnken design.

RUN	A-pH	B-Time（h）	C-Temperature（℃）	D-Liquid-solid Ratio	Yield（%）
1	9.00	2.00	50.00	1.00	71.24
2	10.00	2.50	50.00	1.00	68.49
3	9.00	3.00	50.00	1.00	69.52
4	9.00	2.50	40.00	0.50	66.47
5	9.00	2.00	45.00	0.50	64.32
6	9.00	3.00	45.00	1.50	68.31
7	10.00	2.50	45.00	0.50	65.46
8	9.00	2.50	45.00	1.00	75.01
9	9.00	2.50	45.00	1.00	75.4
10	10.00	2.00	45.00	1.00	70.69
11	9.00	2.50	45.00	1.00	74.83
12	8.00	2.50	50.00	1.00	71.28
13	8.00	2.50	45.00	1.50	70.28
14	9.00	2.50	40.00	1.50	72.51
15	8.00	2.50	45.00	0.50	64.3
16	8.00	3.00	45.00	1.00	72.61
17	9.00	2.50	45.00	1.00	74.99
18	10.00	3.00	45.00	1.00	68.94
19	10.00	2.50	40.00	1.00	73.46
20	9.00	3.00	45.00	0.50	67.89
21	10.00	2.50	45.00	1.50	66.92
22	8.00	2.50	40.00	1.00	72.33
23	9.00	2.50	45.00	1.00	74.97
24	9.00	2.50	50.00	0.50	67.53
25	9.00	3.00	40.00	1.00	73.82
26	9.00	2.00	45.00	1.50	68.88
27	9.00	2.50	50.00	1.50	66.55
28	8.00	2.00	45.00	1.00	69.55
29	9.00	2.00	40.00	1.00	71.73
